# Novel Antioxidant and Antibacterial Injectable Hydrogels Incorporating Clove Oil‐Loaded Mesoporous Bioactive Glass Nanoparticles: A Promising Strategy for Enhanced Bone Regeneration

**DOI:** 10.1002/mabi.202500252

**Published:** 2025-08-19

**Authors:** Andrada‐Ioana Damian‐Buda, Markus Lorke, Aldo R. Boccaccini, Irem Unalan

**Affiliations:** ^1^ Institute of Biomaterials Department of Materials Science and Engineering University of Erlangen–Nuremberg Erlangen Germany

**Keywords:** antibacterial activity, antioxidant activity, bone regeneration, essential oils, injectable hydrogel, mesoporous bioactive glass nanoparticles

## Abstract

Alginate (Alg)—gelatin (Gel)—xanthan gum (Xan) injectable hydrogels incorporating mesoporous bioactive glass nanoparticles (MBGNs) loaded with different concentrations of clove oil (MBGNs+CLV) were developed for bone regeneration applications. The hydrogels exhibited an interconnected network of pores, with their dimensions fulfilling the requirements for bone tissue engineering. MBGNs+CLV were successfully embedded within the Alg‐Gel‐Xan matrix. The presence of CLV in the hydrogel was proven by the total phenolic content (TPC), which was further correlated with the enhanced antioxidant activity, which increased with higher CLV concentrations. Simultaneously, the addition of MBGNs+CLV led to an increase of the hydrogel's Young's modulus, while maintaining its injectability and adhesion properties. Based on the CLV release profile, a burst release was followed by a sustained and controlled release, which was independent of the loaded CLV concentration. The drug release profiles were further correlated with the degradation study, revealing the possible mechanism of drug release. The MBGNs+CLV‐based hydrogel possessed strong antibacterial properties and promoted MC3T3‐E1 pre‐osteoblast cell viability without altering cell morphology. In conclusion, our study highlights the potential of Alg‐Gel‐Xan containing MBGNs+CLV as an antioxidant injectable hydrogel with controlled and sustained CLV delivery, offering a promising strategy for stimulating bone regeneration and addressing bone infection and oxidative stress.

## Graphical abstract

A novel injectable hydrogel composed of alginate–gelatin–xanthan gum incorporating clove oil (CLV)‐loaded mesoporous bioactive glass nanoparticles is developed. The system demonstrates enhanced antioxidant and antibacterial activity, increased Young's modulus, and good injectability. CLV shows a two‐step release profile (burst and sustained release), while in vitro studies confirm the hydrogel biocompatibility, supporting its potential for bone regeneration.

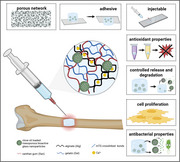

## 1 Introduction

Worldwide, in 2019, the number of cases associated with acute or long‐term symptoms of fractures increased by 70% compared to 1990 [1]. Projections show a doubling of hip fracture cases by 2050, highlighting the urgent need for innovative approaches in bone tissue engineering (BTE) [2]. In this regard, BTE employs two key strategies, which involve the use of preformed scaffolds or injectable scaffolds [3]. While the former involves invasive surgeries for implantation, the latter allows minimally invasive procedures, reducing infection risk and enhancing patient compliance [3, 4]. Moreover, injectable scaffolds can easily fit in any irregularly shaped bone defect [3]. These injectable bone fillers can be further divided into cementitious (e.g., poly (methyl methacrylate) (PMMA), bioactive calcium phosphate (CPC), bone cements, etc.) or hydrogel‐based‐scaffolds (e.g., based on alginate (Alg), gelatin (Gel), poly (ethylene glycol), etc.) [4, 5, 6]. Despite providing optimal mechanical support, traditional bone cements face limitations due to high density and exothermic reactions, impeding their role as carries of cells and growth factors [7]. To address these challenges, recent efforts have focused on developing injectable hydrogels.

Several natural polymers, including Alg [8, 9], Gel [10, 11], or silk [12], have been extensively explored for their potential as injectable hydrogels. Among them, Alg, a natural anionic hydrophilic polysaccharide isolated from brown algae, stands out for the ease with which the hydrogel network is formed [13]. This occurs through non‐covalent crosslinking between the G units present in the Alg backbone and multivalent cations [13]. For instance, Zhang et al. [14]. developed an Alg‐based hydrogel whose injectability was tailored by changing the cation's concentration and type for crosslinking (Ca^2+^, Cu^2+^, Mn^2+^, Fe^3+^, etc.). Similarly, Zhang et al. [15] highlighted the importance of the dynamic ionic bond formation in Alg injectable hydrogels. In their study, they modified Alg with dopamine and crosslinked it with Sr^2+^, resulting in the formation of stable bonds. As a result, an Alg‐based injectable hydrogel with strong adhesive and osteogenic properties was obtained. Despite intensive research on Alg hydrogels, using them alone has proven unsatisfactory from a mechanical point of view [10].

To enhance the overall mechanical performance of Alg hydrogels, researchers are exploring the synergistic blending of Alg with other biopolymers, such as xanthan gum (Xan) [16]. Produced by *Xanthomonas campestris* bacteria, Xan is an anionic exo‐polysaccharide with a repeating sequence of D‐glucose, D‐mannose, and D‐glucuronic acid units at a molar ratio of 2:2:1 [17]. Its unique secondary structure undergoes a transition from a flexible helical conformation to a coiled structure when the solution`s ionic strength decreases or the temperature increases, providing resistance to acidity [18]. Alongside its high resistance to acidity, Xan is degradable, biocompatible, non‐toxic, and promotes cell growth, being already approved by the Food and Drug Administration (FDA) as an additive in food [17, 18]. Simultaneously, these properties make Xan the ideal candidate for controlled drug delivery systems. Moreover, Xan has been found to improve mechanical properties of Alg‐based 3D printed hydrogels, as previously demonstrated by Unalan et al. [16]. More precisely, the authors developed 3D‐printed structures for wound healing applications by blending Alg and Xan and incorporating clove oil (CLV). The results indicated that Xan improved not only the mechanical stability but also the printability of the system without exhibiting cytotoxicity. Similar findings were reported by Cofelice et al. [19], showing a direct correlation between Xan concentration, viscosity, and mechanical properties in the Alg‐Xan system.

While the combination of natural polysaccharides like Alg and Xan holds promise for BTE, their efficacy is hindered by poor cell adhesion due to the lack of specific biomolecular domains that can be easily recognized by cells [20]. In this regard, Gel can be used to enhance the biological properties of hydrogels because it contains the well‐known arginine‐glycine‐aspartic acid (RGD) peptide sequence, which promotes cell attachment and proliferation [21]. Extracted from collagen by acidic or alkaline treatment, Gel has a linear structure with a repetitive Gly‐X‐Y sequence [21, 22]. In addition to its high biocompatibility, degradability, and low toxicity, Gel can be easily degraded by the body without triggering immunogenic responses [21, 22, 23]. Therefore, it is not surprising that pristine or modified Gel has already been investigated as an injectable hydrogel. For example, in the calvarial bone defect model, gelatin‐methacrylate (GelMA)—β‐tricalcium phosphate‐reinforced (TCP) hydrogels showed good integration into the bone, with an enhanced mineralization capacity for the composition containing TCP [24]. Similarly, Wang et al. [25] utilized GelMA combined with Ag‐hydroxyapatite nanoparticles, resulting in composite injectable hydrogels with good cell adhesion, improved mechanical stiffness, enhanced antibacterial activity, and cytocompatibility.

On the other hand, these injectable hydrogels can not only act as void fillers but also be enriched with nanoparticles (Fe_3_O_4_, SiO_2_, Ag, etc.) [12, 26] or bioactive agents [27, 28] to increase their therapeutic capabilities. For instance, by incorporating mesoporous bioactive glass nanoparticles (MBGNs) the mineralization process can be accelerated, while the simultaneous ion release can stimulate specific cellular pathways directly involved in bone regeneration (osteogenesis, angiogenesis, etc.) [29, 30, 31, 32, 33, 34]. Moreover, the tunable mesoporosity, along with the narrow pore size distribution and large surface area, make MBGNs suitable as drug nanocarriers, whose biological activity can be synergistically enhanced by the release of biologically active ions [35, 36, 37]. In a previous study, we have demonstrated the high potential of clove oil (CLV)‐loaded MBGNs in treating bone infections [38]. More precisely, while CLV conferred antioxidant and antibacterial properties to the system, MBGNs acted as an effective loading support, showing sustained and prolonged release for up to 14 days. Our investigation also showed that increasing CLV concentrations enhanced the antioxidant properties without compromising the release profile, cell viability, and morphology.

Therefore, in this study, we aim to develop a complex injectable hydrogel based on Alg‐Gel‐Xan enriched and reinforced with CLV‐loaded MBGNs. To the best of the authors’ knowledge, Alg‐Xan‐Gel injectable hydrogels containing CLV‐loaded MBGNs have not yet been reported in the literature. Herein, Alg is blended with Xan to improve the mechanical properties, while conferring enhanced cell attachment to the system. The incorporation of CLV‐loaded MBGNs imparts antioxidant and antibacterial properties, along with controlled and prolonged drug release capabilities. While the individual effects of CLV, MBGNs, Xan, Gel or Alg have been extensively investigated independently in the literature, the novelty of this study lies in the incorporation of pre‐CLV‐loaded MBGNs into hydrogels. In this way, the physicochemical limitations of CLV, particularly its volatility and hydrophobicity, which typically lead to poor stability, low encapsulation efficiency, and uncontrolled release when incorporated into hydrophilic hydrogels, can be effectively overcome. To explore the impact of CLV concentrations on these properties, two different concentrations of CLV loaded in MBGNs (MBGN+1.5CLV and MBGNs+3CLV) were used. The investigations were focused on the effect of CLV‐loaded MBGNs on the morphology, injectability, and adhesion properties of the hydrogel. The dependency of antioxidant activity and total phenolic content on the CLV concentration was also investigated. Additionally, the correlation between the CLV release and the hydrogel degradation was studied. This relationship is particularly important, especially for the delivery of hydrophobic, volatile natural compounds, whose bioavailability and therapeutic efficacy are often compromised in aqueous environments. Thus, new information about the influence of the hydrophilic hydrogel matrix on the release of the hydrophobic CLV compound can be gained. Finally, antibacterial and cell studies were carried out to confirm the possibility of these systems as highly biocompatible essential oil‐enriched injectable hydrogels for BTE.

## 2 Results and Discussion

### 2.1 Morphological Characterization

**FIGURE 1:**
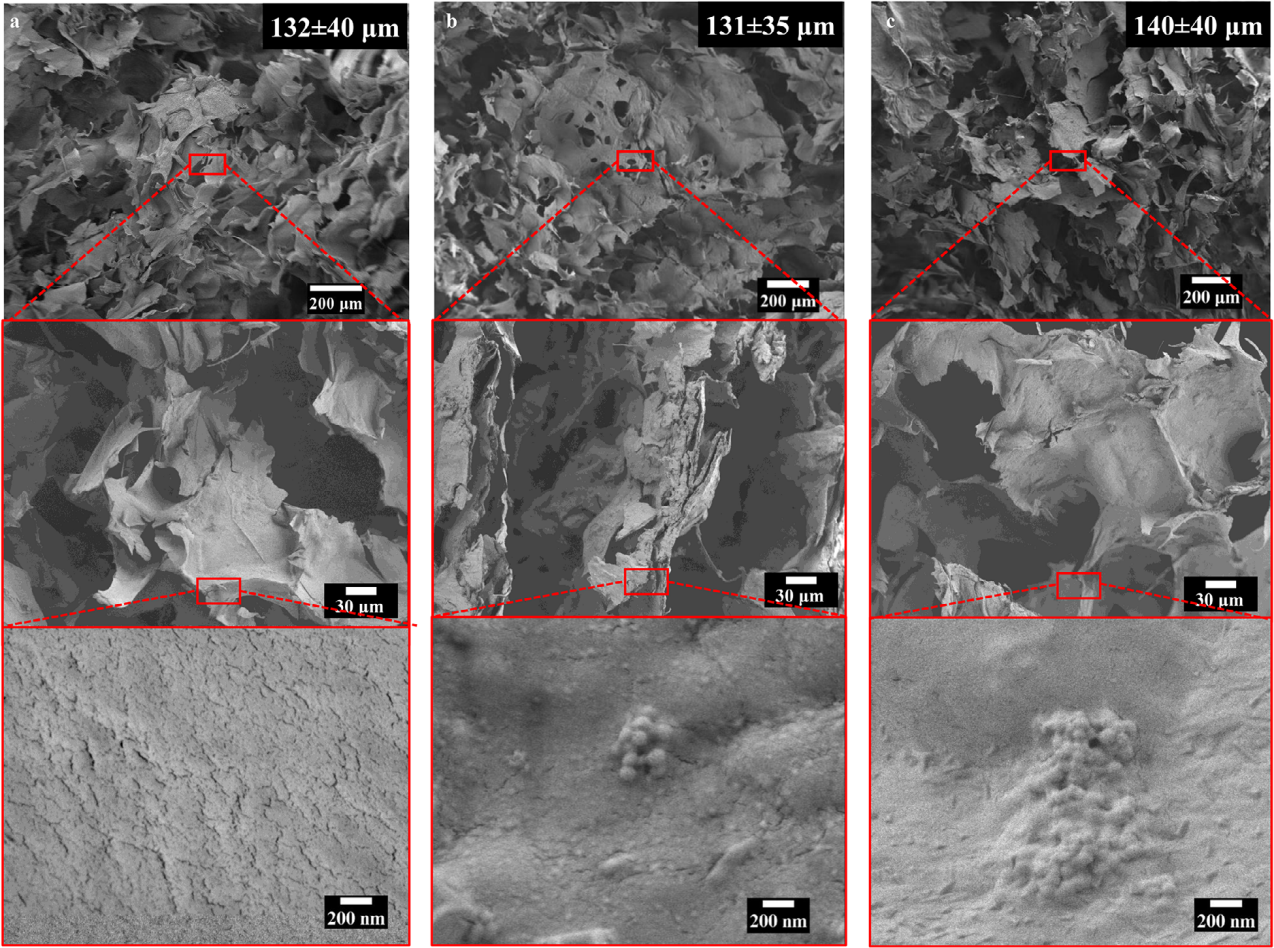
SEM images and average pore size of (a) Alg‐Gel‐Xan, (b) Alg‐Gel‐Xan‐MBGNs+1.5CLV, and (c) Alg‐Gel‐Xan‐MBGNs+3CLV freeze‐dried hydrogels. The top images represent the overall microstructure at low magnification, whereas the bottom images show the detailed surface morphology of the samples. The mean pore size and standard deviation were determined after measuring 50 pores for each hydrogel, which were cast into cylindrical moulds (10 mm diameter, 4 mm height).

After freeze‐drying the hydrogels, the scaffolds` morphology was examined by SEM. The low‐magnification images (Figure 1) revealed, for all samples, a similar porous structure with an interconnected pore network, essential for facilitating cell migration and blood vessel formation [39, 40]. The pores had diameters ranging from 100 to 200 µm, being within the optimal range (100–500 µm) required for BTE applications [39]. Importantly, no significant differences in terms of pore size were noticed among the different compositions. The detailed images of the surfaces revealed a rough surface with ridges and valleys, most probably due to the interactions between the polymer chains during the freeze‐drying process. Furthermore, for Alg‐Gel‐Xan‐MBGN+1.5CLV and Alg‐Gel‐Xan‐MBGNs+3CLV, CLV‐loaded MBGNs covered with a thin layer of polymer could be identified, proving the successful integration of the MBGNs in the hydrogels. These results are consistent with data reported by Wu et al. [41], who incorporated melatonin‐loaded MBGNs in Alg hydrogels for intervertebral disc regeneration, without affecting the morphology of the samples. Similar to our findings, Kohoolat et al. [42] showed that the presence of Cu‐doped BG particles in a sodium Alg‐carboxymethyl cellulose hydrogel did not alter either the pore interconnectivity or size. The same optimal pore size range was obtained when Ce‐doped BG particles were incorporated in an Alg/Gel scaffold [43]. However, in contrast to these observations, Naruphontjirakul et al. [44] reported a significant reduction in pore size when Zn‐Sr‐co‐doped BG nanoparticles were present in an Alg hydrogel, which was attributed to the possible crosslinking between Alg chains and BG nanoparticles, as a consequence of the dissolution products released from the BG nanoparticles.

### 2.2 Chemical Characterization

**FIGURE 2:**
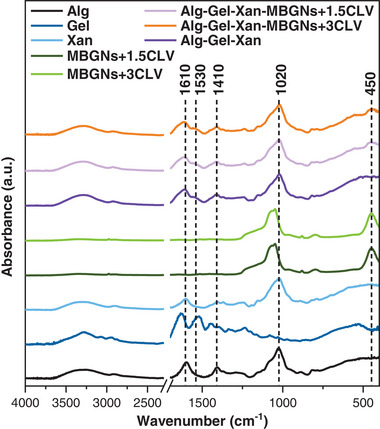
ATR‐FTIR spectra of Alg, Gel, Xan, MBGNs+1.5CLV, MBGNs+3CLV, Alg‐Gel‐Xan, Alg‐Gel‐Xan‐MBGNs+1.5CLV and Alg‐Gel‐Xan‐MBGNs+3CLV. The bands highlighted in the figure are discussed in detail in the corresponding section.

The ATR‐FTIR analysis was performed to determine the presence of Alg, Gel, Xan, MBGNs+1.5CLV, and MBGNs+3CLV in the samples. As depicted in Figure 2, the typical peaks of Alg, Gel, and Xan were identified across all samples. More precisely, the C─O─C bond stretching in the cyclic ether bridge of Alg was identified at 1020 cm^−1^, while the band at 1410 cm^−1^ might correspond to the symmetric (COO^−^) group stretching in Alg [45]. However, for both bands (1020 and 1410 cm^−1^), the spectral signal coming from Alg and Xan overlaps, suggesting potential signal contributions from both compounds. Therefore, the band at 1410 cm^−1^ could also come from the symmetric stretching of the carboxylic group in Xan [46], whereas the one at 1020 cm^−1^ could be assigned to the (C─O─C) stretching bond in Xan [47]. The specific bands of Gel, amine I, and N‐H deformation for amide II, were identified at 1610 and 1530 cm^−1^, respectively [48]. Nevertheless, the band at 1610 cm^−1^ could also arise from the asymmetric (COO^−^) stretching in Alg [45] or the deformation of (C═O) bonds from Xan [47]. Compared to the spectrum of Gel, Xan, and Alg, these bands were shifted to higher wavenumbers for Alg‐Gel‐Xan, Alg‐Gel‐Xan‐MBGNs+1.5CLV, and Alg‐Gel‐Xan‐MBGNs+3CLV. This might be due to the overlapping signals coming from the individual Alg, Gel, and Xan, leading to a broadening and slight shift of the absorbance peaks. Another possible explanation could be the crosslinking with Ca^2+^, as previously reported [16]. On the other hand, the absence of significant changes in the wavenumber of the carbonyl stretching regions suggests that no covalent bonds were formed between Alg and Gel. Instead, Alg, Gel, and Xan could interact by non‐covalent bonding, such as electrostatic interactions, hydrogen bonding, and physical entanglement [49]. Additionally, for the MBGNs+CLV‐containing hydrogels, an absorption peak from the Si‐O‐Si rocking vibration was identified at 450 cm^−1^ [32, 34]. However, characteristic bands of CLV could not be seen, most probably because of its low concentration in the hydrogels.

### 2.3 Injectability and Adhesion Properties

**FIGURE 3:**
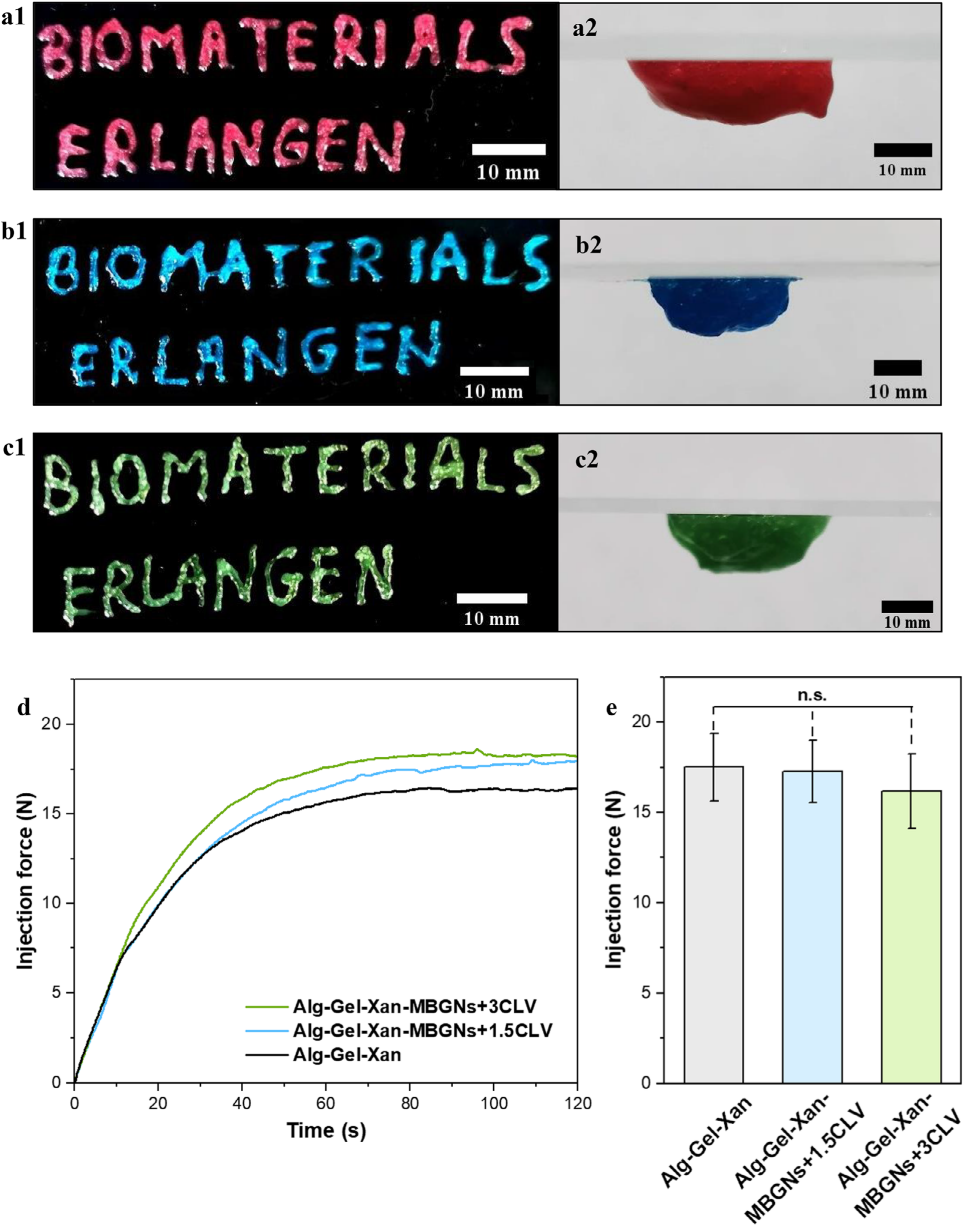
Representation of injectability and adhesion test results for (a1‐2) Alg‐Gel‐Xan, (b1‐2) Alg‐Gel‐Xan‐MBGN+1.5CLV, and (c1‐2) Alg‐Gel‐Xan‐MBGN+3CLV; (d) Injection force as a function of time along with (e) the average injection force. The experiments were conducted in five replicates, and the results are expressed as the mean value ± standard deviation (SD). Statistical analysis was performed using one‐way ANOVA followed by Bonferroni post hoc analysis, data were assessed for homogeneity of variance using Levene's test, as implemented in Origin 2023. (**n.s.—**not significant).

During the injection process of the hydrogel into the bone defect, it is crucial that the material flows continuously and uniformly. Therefore, the hydrogels` injectability was assessed by manually and constantly applying pressure to extrude the hydrogels through a syringe. Figure 3a1–c1 shows that the quality of the extruded material was not affected by the addition of MBGNs+1.5CLV and MBGNs+3CLV to the hydrogel. In all cases, the hydrogels exhibited an easy, continuous, and uniform extrusion from the needle, showing good injectability properties. These qualitative observations were further supported by measuring the injection force, which provides detailed insights into the mechanical behaviour of the hydrogel during extrusion. As shown in Figure 3d, the injection force‐time profiles of all types of hydrogels exhibited a similar two‐phase pattern. Initially, the force increased linearly with time as the pressure increased in the syringe, reflecting the force required to overcome the static friction and initial flow resistance to enable hydrogel extrusion. This phase was followed by a plateau phase, during which the hydrogel was extruded at a constant rate under pressure [50]. Moreover, the smooth force profile observed for the MBGNs‐containing hydrogels suggests that the nanoparticles were well dispersed in the material [50]. Quantitatively, the average injection force calculated for all samples was below 20 N (Figure 3e), a threshold reported as optimal for ease of handling and patient comfort [51]. Importantly, the incorporation of MBGNs+1.5CLV and MBGNs+3CL did not significantly alter the injectability of the hydrogels. Notably, our results are in agreement with those reported by Guan et al. [52], who used nanohydroxyapatite and nano BGs as inorganic fillers for Alg hydrogels. On the other hand, Pontremoli et al. [53] conducted a study assessing the effect of various synthesis methods of Ce‐doped BGs on the injectability properties of Poloxamer 407 hydrogel. Their findings proved that the injectability of the hydrogels can be finely tuned by using different types of particles, as they had different sizes, which leads to different types of interactions with the polymer chains.

In addition to injectability, the hydrogels should be able to adhere to the surrounding tissue. Thus, adhesion tests were performed by casting the hydrogels on glass slides, flipping them around, and observing signs of flow. After 15 min of testing, for all samples, there were no signs of hydrogels flowing from the glass slide, proving excellent adhesivity even after the addition of the nanoparticles as depicted in Figure 3 a2–c2. This phenomenon can be explained by the hydrogen bonds formation between the glass surface, which is rich in (─OH) groups, and the hydrophilic groups present in the hydrogel (─COOH, ─NH_2_, ─OH, etc.). The Gel, derived from collagen, contains a wide range of side‐chain functional groups, with the main three constituting amino acids, glycine, proline, and hydroxyproline, which are rich in (─COOH), (‐NH_2_), and (─OH) groups. Similarly, Alg and Xan are both abundant in (─OH) and (─COOH) groups, further enhancing the material`s ability to form hydrogen bonds with (OH) rich surfaces. The synergistic combination of these functional groups ensures a strong and stable adhesion to hydrophilic surfaces. Consistent with our results, the same type of MBGNs, but without drug loading, were used together with Gel and dialdehyde starch to develop a strong adhesive hydrogel for wound healing [54]. Their results proved the strong adhesive properties of the nanocomposites, with a significantly higher adhesion strength after MBGN addition. This enhancement was attributed to the molecular interactions between MBGNs, the hydrogel, and the soft or hard tissue substrates used, confirming the connecting abilities of MBGNs. In a recent study by Yang et al. [55], the incorporation of nano BGs in dopamine‐modified Gel and oxidized hyaluronan matrix did not compromise the hydrogel adhesive properties on various substrates. Moreover, changes in BG composition did not alter the adhesiveness, the final hydrogel being able to hold itself on glass, metal, bone, and polytetrafluoroethylene substrates.

### 2.4 Mechanical Properties

The hydrogels' mechanical properties, especially the stiffness, in contact with cells, are one of the pivotal cues for cellular fate and development [56, 57]. Therefore, the Young´s modulus was investigated by performing compression tests.

**FIGURE 4:**
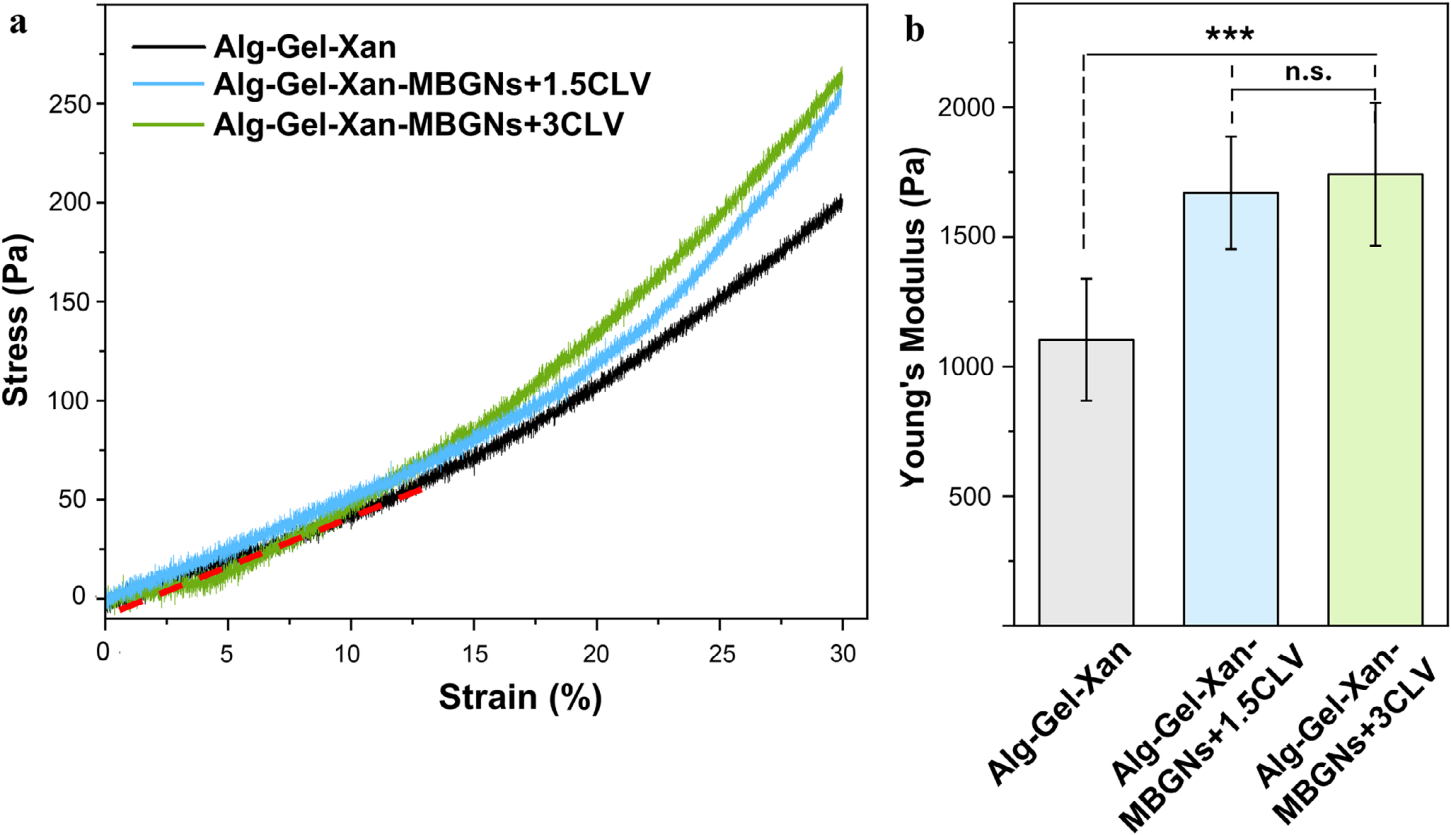
(a) Stress‐strain curves and (b) average Young's modulus values for Alg‐Gel‐Xan, Alg‐Gel‐Xan‐MBGNs+1.5CLV, and Alg‐Gel‐Xan‐MBGNs+3CLV. Hydrogels were cast into cylindrical molds (10 mm diameter, 4 mm height). The dashed red line in (a) represents the modulus calculation region. The experiments were conducted in quadruplicate, and the results are expressed as the mean value ± standard deviation (SD). After performing statistical analysis using one‐way ANOVA followed by Bonferroni post hoc analysis, data were assessed for homogeneity of variance using Levene's test, as implemented in Origin 2023, the asterisks denote significant differences (^***^ *p* < 0.001, **n.s**.—not significant).

Figure 4a,b shows typical stress‐strain curves and the averaged Young´s moduli for the three hydrogel compositions tested. The MBGNs containing compositions exhibit a stress‐induced stiffening after an almost linear region until 10% strain, which was the same for all hydrogels. Within this region, the Young's modulus was calculated and the differences between the hydrogels became clearer. While the pure Alg‐Gel‐Xan hydrogel shows significantly lower stiffness than the MBGNs‐containing samples, increasing the CLV content in the MBGNs did not induce significant changes. The observed increase in Young's modulus in the presence of MBGNs+1.5CLV or MBGNs+3CLV can be attributed to electrostatic interactions between the hydrogel components and the surface of the CLV‐loaded nanoparticles. In previous work, we have shown that MBGNs+1.5CLV were positively charged, while MBGNs+3CLV had a negative surface charge [38]. Thus, hydrogen bonds could form between the positively charged amino groups in Gel and the surface of the MBGNs+3CLV. At the same time, the positively charged MBGNs+1.5CLV could establish hydrogen bonds with the carboxyl (COO^−^) groups in Alg [58, 59, 60]. Furthermore, the release of Ca^2+^ ions from the MBGNs could facilitate the crosslinking of Alg, thereby enhancing the mechanical stability of the constructs [61].

Monavari et al. [59] reported similar observations regarding the increased stiffness of the hydrogels when incorporating lysozyme‐loaded Ce‐doped MBGNs in alginate dialdehyde‐Gel (ADA‐Gel) matrix. This improvement was attributed to the potential formation of hydrogen bonds between the negatively charged particles and the positively charged amino groups in Gel. Our findings are also in agreement with the previous work from Chakraborty et al. [62], who examined the effect of different filler concentrations on the mechanical behaviour of hydrogels, showing that nanoparticle‐laden hydrogels benefit from the additional reinforcing structure, allowing stress to be distributed onto the stiffer particles, unlike in the pure hydrogel. The loading capacity of the drug has no significant influence on the reinforcing effect of the nanoparticles, in contrast to an increase in the amount of particles. Such an effect could only be caused by the presence of divalent ions in the drug [62, 63]. On the other hand, Bider et al. [64] explored the influence of ferulic acid (FA), a phytotherapeutic agent, or/and human platelet lysate (HPL), a mixture of plasma proteins and growth factors, on the stiffness of ADA‐Gel hydrogels. While the FA containing compositions (ADA‐Gel‐FA) showed a higher effective modulus, the interaction with HPL led to a reduction in stiffness, with a stronger decrease observed with increasing HPL content. Combining HPL and ADA‐Gel‐FA also decreased the mechanical properties with respect to the reference ADA‐Gel. The positive impact of FA on the effective modulus can be attributed to its ability to form ester bonds with Gel and ADA, reinforcing the hydrogel matrix. In contrast, the large molecular size of HPL and complex composition could prevent the formation of crosslinking bonds within the hydrogel network, thus reducing its structural integrity. Interestingly, while the simultaneous incorporation of FA and HPL in the same matrix did not enhance the mechanical properties, the opposite behaviour was observed when FA was combined with MBGNs in the same matrix material [65]. This effect likely arises from the in situ ability of the MBGNs to act as a crosslinker for the hydrogel matrix, by directly forming electrostatic interactions with the positive regions of the polymer chains or, indirectly, by releasing Ca^2+^, which could crosslink the Alg network.

### 2.5 In Vitro Release and Degradation Studies

**FIGURE 5:**
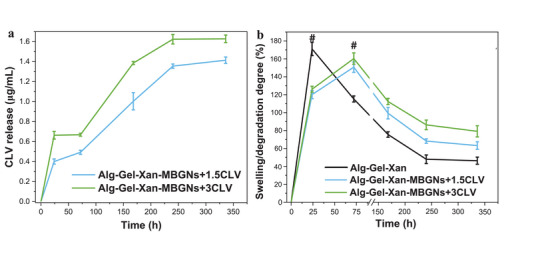
(a) In vitro cumulative release profile of CLV and (b) degradation profile of Alg‐Gel‐Xan, Alg‐Gel‐Xan‐MBGNs+1.5CLV, and Alg‐Gel‐Xan‐MBGNs+3CLV determined based on weight change following Equation (1). Hydrogels were cast into cylindrical molds (10 mm diameter, 4 mm height). The ‘#’ symbol shows the maximum swelling degree for each type of hydrogel. The experiments were conducted in triplicate, and the results are expressed as the mean value ± standard deviation (SD).

The development of hydrogels with the ability to release active compounds has gained significant interest recently due to the potential of achieving localized and controlled drug release, which can minimize systemic side effects and enhance drug efficacy at lower doses. Thus, in our study, we investigated the release of CLV from the hydrogel, aiming at an initial burst release to effectively mitigate the risk of infection, followed by a sustained release over the long term. The release kinetics of CLV is directly influenced by the swelling/degradation behavior of the hydrogels. Therefore, the in vitro CLV release profiles were discussed in parallel with the swelling/degradation results (Figure 5).

In the first 24 h, both Alg‐Gel‐Xan‐MBGNs+1.5CLV and Alg‐Gel‐Xan‐MBGNs+3CLV exhibited an initial burst release of CLV. At the same time, the degradation study revealed an increase in cell culture medium uptake, indicative of pronounced hydrogel swelling. Correlating these findings, the initial burst release might be attributed to the increased swelling, which could create more diffusion pathways within the hydrogel matrix. This, in turn, allows CLV molecules, which are loosely bonded to the MBGNs, to diffuse more easily out of the material. Between 24 and 72 h, the concentration of the released CLV remained constant, whereas the water absorption capacity of the hydrogels continued to increase, but at a slower pace compared to the previous phase. Despite reaching a higher swelling degree, the drug was released at a constant rate, a difference that could arise from the fact that CLV molecules are electrostatically loaded into the nanocarrier support, which could lead to a delay in release. During the next release phase (72—240 h), the degradation of the hydrogels resulted in an increased concentration of released CLV. This phenomenon can be attributed to several simultaneous processes taking place within the hydrogel matrix. First, as the degradation progresses, the porosity of the hydrogels increases owing to the loosening of the polymer network. The higher porosity provides additional pathways for the diffusion of drug molecules. Simultaneously, the degradation process might result in the breakdown of the polymer chains within the hydrogels. This mechanism could lead to erosion and dissolution of the material, thereby facilitating the diffusion of the drug molecules out of the material. After 240 h, both the CLV concentration and the weight of the samples remained constant, proving a controlled and sustained CLV release up to 336 h.

Notably, both hydrogels exhibited similar CLV release and degradation profiles, independent of the amount of CLV loaded within the MBGNs. As expected, at all testing points, a higher amount of CLV was released from Alg‐Gel‐Xan‐MBGNs+3CLV compared to Alg‐Gel‐Xan‐MBGNs+1.5CLV because the MBGNs+3CLV contained a higher dose of loaded CLV. Monavari et al. [66] also investigated the correlation between degradation and drug release. They used MBGNs as nanocarriers for icariin, which were further embedded in ADA‐Gel hydrogels. Consistent with our findings, they showed that the release of icariin from the constructs was influenced not only by the degradation kinetics of the hydrogel, but also by the drug release from the MBGNs. The release of icariin from the hydrogels showed an initial burst release, followed by a sustained and prolonged release for up to 35 days. At the same time, the weight loss of the hydrogels was significantly reduced in the presence of the loaded MBGNs, proving the stabilizing effect of the MBGNs on the hydrogels. In another study, Bider et al. [67] explored the effect of using different concentrations of ferulic acid (0.1%, 0.15% and 0.2% w/v) incorporated in ADA‐Gel scaffolds on the drug release and scaffold degradation. The results highlighted a similar release and degradation kinetics for all the hydrogels, despite using different concentrations of added ferulic acid. More precisely, an initial burst release followed by a continuous and sustained release for up to 28 days was reported. Unexpectedly, for the highest concentration of ferulic acid, the release was slower compared to the middle value, most probably due to the poor solubility of the drug at high concentrations in non‐polar solvents. In another study, CLV was emulsified and embedded directly into a chitosan‐oxidized pullulan hydrogel at two different concentrations (1 and 5% w/w) [68]. In line with our results, CLV was released in two phases, with an initial burst release following 7 h, followed by a continuous release for up to 50 h. Despite these promising observations, our Alg‐Gel‐Xan‐MBGNs+1.5CLV and Alg‐Gel‐Xan‐MBGNs+3CLV showed a more prolonged CLV release. This observation can be attributed to the high capacity of the MBGNs not only for drug loading, but also to their ability to control and ensure a sustained release over long periods of time. In line with our observations, Saha et al. [69] reported the prolonged release of CLV from gelatin–chitosan cryogels up to 14 days, with an initial burst and uncontrolled release lasting 5 days, a process attributed to the drug released from the surface of the scaffold.

Regarding the degradation study, in contrast to Alg‐Gel‐Xan, for which the maximum swelling degree was observed after 24 h of incubation, the Alg‐Gel‐Xan‐MBGNs+1.5CLV and Alg‐Gel‐Xan‐MBGNs+3CLV reached the maximum water uptake after 72 h. This difference might be a consequence of the presence of the MBGNs in the polymer matrix, which could prevent the diffusion of the solvent molecules, thereby delaying the swelling process. Additionally, the starting point of the degradation for Alg‐Gel‐Xan‐MBGNs+1.5CLV and Alg‐Gel‐Xan‐MBGNs+3CLV was observed only after 3 days compared to 1 day for Alg‐Gel‐Xan group. This delay might come from the release of Ca^2+^ from the MBGNs, which could crosslink the Alg, thus slowing down the degradation process. Besides ensuring an indirect release of crosslinking ions that prevents the fast degradation of the material, MBGNs can also enhance the degradation resistance by acting as a direct crosslinker, as presented by Monavari et al. [66]. The authors have investigated the effects of incorporating icariin‐loaded MBGNs into the ADA‐Gel matrix on the degradation behaviour of the materials. Initially, icariin‐containing hydrogels showed a higher mass loss compared to ADA‐Gel, most likely due to the release of icariin from the matrix. However, after 28 days of incubation, the drug‐containing hydrogels exhibited a lower mass loss with respect to the control ADA‐Gel group. This effect was ascribed to both the release of Ca^2+^ ions, which promote in situ crosslinking of Alg, and the direct electrostatic interactions formed between the negatively charged surface of the MBGNs and the positively charged functional groups present in the polymer chain. A similar delay in degradation was reported by Bider et al. [65], following the addition of 0.1% (w/v) MBGNs into the ADA‐Gel hydrogel. Contrary to expectations, increasing the MBGNs content to 0.5% (w/v) did not further enhance the effective modulus of the hydrogels. This result was explained by the formation of nanoparticle agglomerates at these concentrations, which act as weak points and disrupt the hydrogel's network. Both studies are in agreement with our findings, highlighting the beneficial role of MBGNs as direct and indirect crosslinking agents for Alg‐Gel‐based hydrogel systems.

### 2.6 Total Phenolic Content and Antioxidant Activity

In healthy bone, reactive oxygen species (ROS) ensure the balance between the activity of osteoclasts and osteoblasts, but under pathological conditions, the levels of ROS are increased, leading to oxidative stress and finally disrupted bone homeostasis [70]. To tackle this problem, CLV, a known naturally derived antioxidant compound, was loaded into MBGNs, as shown in our previous work [38], and it was incorporated into the hydrogel in the present study.

**FIGURE 6:**
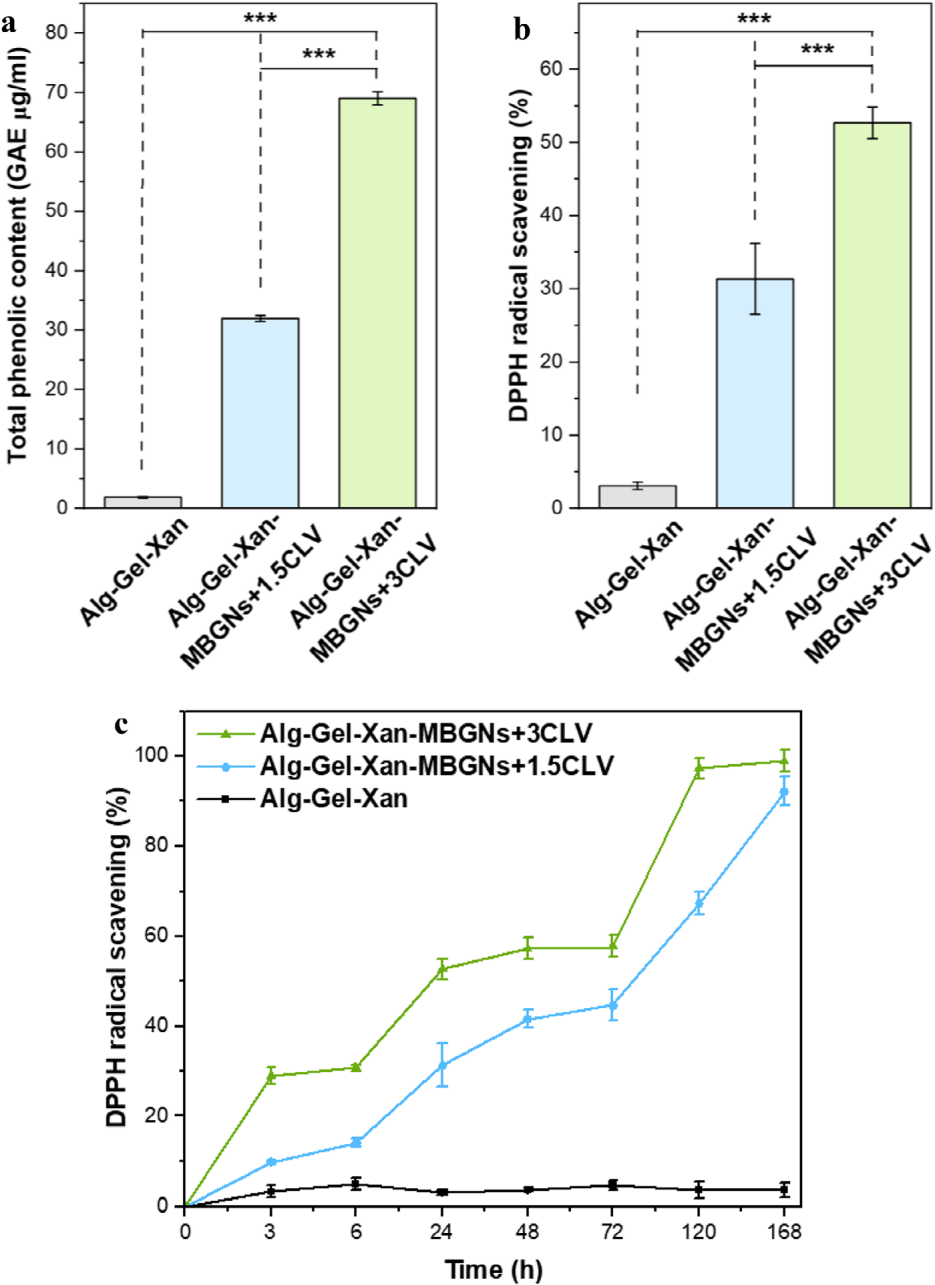
(a) TPC and (b) DPPH radical scavenging activities of Alg‐Gel‐Xan, Alg‐Gel‐Xan‐MBGNs+1.5CLV, and Alg‐Gel‐Xan‐MBGNs+3CLV; (c) DPPH radical scavenging activity profile after 1, 3, 6, 24, 72, 120, and 144 h. Hydrogels were cast into cylindrical molds (10 mm diameter, 4 mm height). The experiments were conducted in triplicate, and the results are expressed as the mean value ± standard deviation (SD). After performing statistical analysis using one‐way ANOVA followed by Bonferroni post hoc analysis, data were assessed for homogeneity of variance using Levene's test, as implemented in Origin 2023, the asterisks denote significant differences (^***^ *p* < 0.001). /(GAE: gallic acid equivalents).

The TPC analysis was conducted to quantitatively assess the amount of CLV present in the materials. As depicted in Figure 6a, for Alg‐Gel‐Xan‐MBGNs+1.5CLV and Alg‐Xan‐MBGNs+3CLV, a significantly higher concentration of phenol compound was recorded compared to Alg‐Gel‐Xan due to eugenol, which is the main compound of CLV. Additionally, this result confirmed the successful integration of CLV in the hydrogels. Moreover, Alg‐Gel‐Xan‐MBGNs+3CLV showed a significantly higher TPC than Alg‐Gel‐Xan‐MBGNs+1.5CLV, a difference that can be attributed to the double amount of CLV loaded into MBGNs, as we have demonstrated in a previous study [38].

As TPC is directly linked to the antioxidant activity, further DPPH radical scavenging tests were carried out (Figure 6b). The results revealed that the Alg‐Gel‐Xan‐MBGNs+1.5CLV and Alg‐Xan‐MBGNs+3CLV hydrogels demonstrated a significantly higher antioxidant activity than Alg‐Gel‐Xan. Between them, the highest DPPH radical scavenging activity was recorded for the Alg‐Gel‐Xan‐MBGNs+3CLV group. This difference can be explained by the higher amount of CLV loaded into MBGN+3CLV compared to MBGNs+1.5CLV. Similar to the TPC results, DPPH radical scavenging activity demonstrated a stronger antioxidant activity for a higher CLV concentration. Furthermore, to evaluate the sustained antioxidant properties of the hydrogels, DPPH radical scavenging activity was assessed at different time points over an extended period of time (Figure 6c). As expected, the Alg‐Gel‐Xan hydrogel, which did not contain antioxidant compounds, showed no changes in the radical scavenging activity at any testing time. In contrast, both Alg‐Gel‐Xan‐MBGNs+1.5CLV and Alg‐Gel‐Xan‐MBGNs+3CLV scaffolds demonstrated a gradual and time‐dependent increase in antioxidant activity, suggesting a gradual release of CLV from the composite systems. Among these two samples, Alg‐Gel‐Xan‐MBGNs+3CLV showed superior antioxidant properties, which can be directly correlated with the higher amount of CLV released (Figure 5a). Furthermore, the maximum antioxidant activity (∼100%) was obtained for Alg‐Gel‐Xan‐MBGNs+3CLV after 5 days, whereas the hydrogel containing MBGNs+1.5CLV reached the saturation point after 7 days. This difference is in agreement with the previous CLV release study (in Section 2.5), where it was shown that CLV was released at a lower concentration from the Alg‐Gel‐Xan‐MBGNs+1.5CLV compared to Alg‐Gel‐Xan‐MBGNs+3CLV. While the DPPH assay is well‐suited for assessing the antioxidant properties of CLV, additional assays (such as 2,2‐azino‐bis(3‐ethylbenzothiazoline‐6‐sulphonic acid) or ferric reducing antioxidant power (FRAP) assays) could provide complementary information about the antioxidant capacity and reducing potential of the materials. These assays should be considered in future studies to comprehensively assess the antioxidant performance.

Our findings are in agreement with former investigations, where it was demonstrated that the incorporation of specific biologically active compounds into hydrogels confers or even enhances their antioxidant activity. For instance, Unalan et al. [16] showed that the TPC and antioxidant activity increased for higher concentrations of CLV, an essential oil that was incorporated into Alg‐Xan 3D‐printed scaffolds. Consistent with these results, Bider et al. [67] proved that the ADA‐Gel‐FA hydrogels` phenolic content increased with higher FA concentration and incubation time. These results were further correlated with the enhanced antioxidant properties achieved, as demonstrated by the increase in DPPH radical scavenging activity with increasing FA content [67]. In another study, the researchers illustrated that increasing the concentration of lignin NPs from 1 to 3 wt.% in polyvinyl alcohol‐chitosan hydrogel led to a slight increase in antioxidant activity [71]. Furthermore, Bai et al. [72] developed a novel system based on protocatechualdehyde complexed with Zn NPs and guar gum. The presence of metallic‐polyphenolic NPs conferred significantly stronger antioxidant properties to the hydrogel compared to those of pure guar gum hydrogel. In a similar approach, Yang et al. [73] proved that combining ZnO NPs and tannic acid in a modified silk fibroin hydrogel resulted in superior antioxidant activity. In contrast to these studies, where phenolic compounds were directly incorporated into different materials, our approach proves that despite loading phytotherapeutic agents into MBGNs, which could potentially delay or mitigate this effect, the hydrogels still exhibited strong antioxidant properties. This might come as a consequence of the effective release of CLV in the first hours of incubation, as presented in Section 2.5.

### 2.7 Antibacterial Activity

**FIGURE 7:**
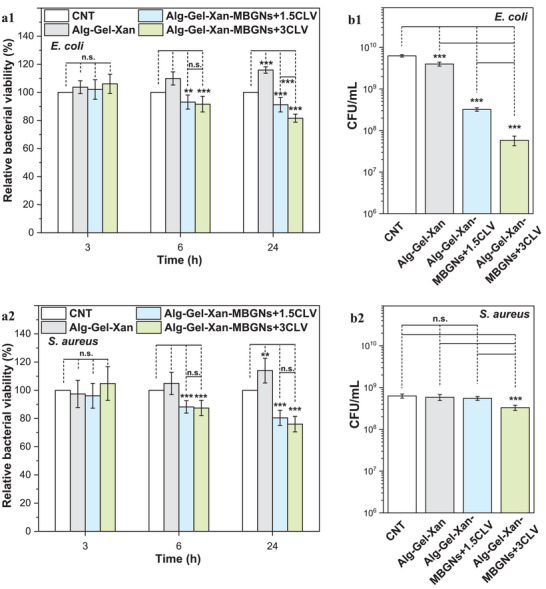
(a1‐2) Relative bacterial viability and (b1‐2) CFU for (a1, b1) *E. coli* (Gram‐negative) and (a2, b2) *S. aureus* (Gram‐positive) of Alg‐Gel‐Xan, Alg‐Gel‐Xan‐MBGNs+1.5CLV, and Alg‐Gel‐Xan‐MBGNs+3CLV after 3, 6, and 24 h of incubation. Hydrogels were cast into cylindrical molds (10 mm diameter, 4 mm height). The experiments were conducted in triplicate, and the results are expressed as the mean value ± standard deviation (SD). After performing statistical analysis using one‐way ANOVA followed by Bonferroni post hoc analysis, data were assessed for homogeneity of variance using Levene's test, as implemented in Origin 2023; the asterisks denote significant differences (^**^ *p* < 0.01,^***^ *p* < 0.001, **n.s.—**not significant).

The incorporation of CLV in the hydrogels was not only aimed at enhancing the antioxidant activity, but also to impart antibacterial properties, crucial for minimizing the development of bone infections (osteomyelitis). Therefore, the antibacterial properties of the hydrogels were evaluated against Gram‐negative (*E. coli*) and Gram‐positive (*S. aureus*) bacteria (Figure 7).

For both bacterial strains, similar trends were observed across all samples. More precisely, after 3 h of incubation, no significant changes in the relative bacterial viability were observed. The initial absence of antibacterial activity might be due to the slow release of CLV, as discussed in Section 2.5. This slow release could result in CLV concentrations falling below the minimum antibacterial concentration threshold. However, after 6 h, Alg‐Gel‐Xan‐MBGNs+1.5CLV and Alg‐Gel‐Xan‐MBGNs+3CLV exhibited significant antibacterial activity against both *E. coli* and *S. aureus* compared to the control and Alg‐Gel‐Xan. This antibacterial effect can be attributed to CLV's presence in the material, leading to the disruption of the bacterial cell wall and membrane, accompanied by irreversible changes in the intracellular components [74]. Similar reductions in the bacterial viability for Alg‐Gel‐Xan‐MBGNs+1.5CLV and Alg‐Gel‐Xan‐MBGNs+3CLV were also observed after 24 h. Nevertheless, the bacterial viability in the Alg‐Gel‐Xan group was significantly higher relative to the control, most probably due to the release of the Alg and Gel, which can serve as nutrients for bacterial growth [75]. Even if comparable antibacterial activities were achieved for both strains, only for *E. coli*, the Alg‐Gel‐Xan‐MBGNs+3CLV significantly reduced viability compared to Alg‐Gel‐Xan‐MBGNs+1.5CLV. This enhancement in antibacterial activity could be attributed to the higher CLV concentration present and released from the material. These findings are further supported by the results of the colony‐forming assay (CFU). For *E.coli*, both CLV‐containing hydrogels significantly reduced the number of colonies compared to the control and the plain hydrogel, with a stronger effect for Alg‐Gel‐Xan‐MBGNs+3CLV. In the case of *S. aureus*, only the Alg‐Gel‐Xan‐MBGNs+3CLV hydrogel demonstrated a statistically significant reduction in CFU with respect to all other groups. These results are in line with the relative bacterial viability results (Figure 7 a1,2). Additionally, these observations are consistent with previous results reported for CLV‐incorporated electrospun nanofibers, proving that CLV is more effective against *E. coli* than *S. aureus* [76].

The same direct dependency between CLV concentration and antibacterial activity against both *E. coli* and *S. aureus* was reported by Unalan et al. [16], who developed Alg‐Xan hydrogels with varying CLV concentrations. In addition, in a related study utilizing the same bacterial strains, the presence of CLV in Gel‐chitosan cryogels conferred strong antibacterial properties to the materials [69]. Similarly, Suflet et al. [68] compared the antibacterial properties of oxidized pullan‐chitosan hydrogels containing different concentrations of CLV, against *E. coli* and *S. aureus*. Similar to our results, the antibacterial activity increased with higher CLV concentrations, being more effective against *E. coli* than *S. aureus*. On the other hand, other investigations have been focused on the effect of using nanocarriers containing antibacterial compounds in hydrogels [59, 77]. For instance, Monavari et al. [59] developed 3D‐printed scaffolds based on Gel reinforced with lysozyme‐loaded Ce‐doped MBGNs and gentamicin. Despite lysozyme's inherent antibacterial properties, no notable decrease in bacterial viability was observed relative to the hydrogel containing only gentamicin. The authors ascribed this phenomenon to the gradual release of lysozyme from the MBGNs. In contrast, Mittal et al. [77] demonstrated that incorporating CLV‐loaded silica nanoparticles in poly(hydroxybutyrate) films led to a stronger and longer‐lasting antibacterial effect than the simple CLV loading in the material, attributed to the controlled release facilitated by the drug carrier.

**FIGURE 8:**
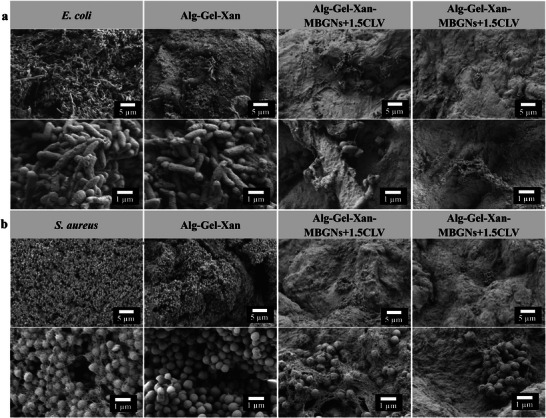
(a) SEM images of *E. coli* and (b) *S. aureus* after 24 h of incubation in different groups: control (bacterial suspension), Alg‐Gel‐Xan, Alg‐Gel‐Xan‐MBGNs+1.5CLV, and Alg‐Gel‐Xan‐MBGNs+3CLV.

On the other hand, the qualitative adherence of *S. aureus* and *E. coli* on the hydrogel surface was investigated by SEM analysis, and the results are shown in Figure 8. SEM images of the samples following the antibacterial tests revealed a lower density of bacterial colonies attached to the surface of CLV‐containing MBGN hydrogels, further corroborating the quantitative data. To precisely understand the antibacterial mechanisms, subsequent investigations should focus on elucidating the specific molecular and biophysical mechanisms behind the CLV‐mediated antibacterial activity.

### 2.8 In Vitro Cytocompatibility and Cell Morphology

**FIGURE 9:**
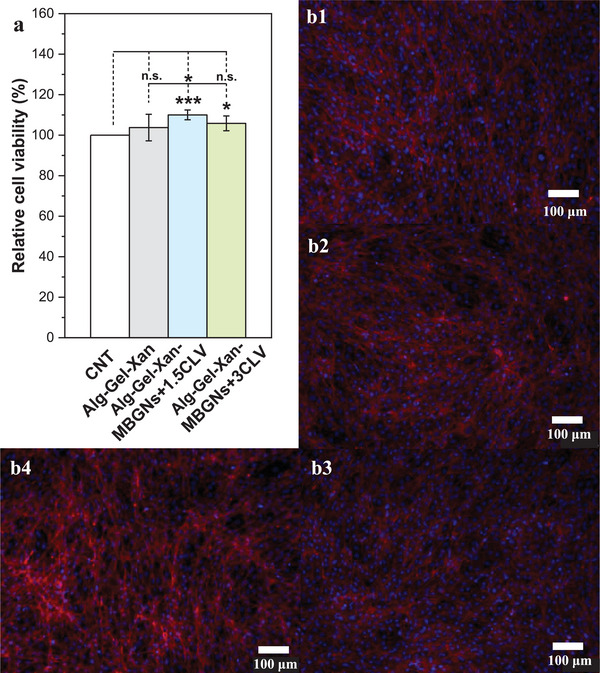
(a) Relative cell viability of MC3T3‐E1 pre‐osteoblast cells after 48 h of incubation in the presence of the hydrogels. Fluorescence microscopy images of MC3T3‐E1 pre‐osteoblast cell nuclei and cytoskeleton after 48 h of incubation with (b1) control, (b2) Alg‐Gel‐Xan, (b3) Alg‐Gel‐Xan‐MBGNs+1.5CLV, and (b4) Alg‐Gel‐Xan‐MBGNs+3CLV (red‐F‐actin and blue‐nuclei). The experiments were conducted in quadruplicate, and the results are expressed as the mean value ± standard deviation (SD). After performing statistical analysis using one‐way ANOVA followed by Bonferroni post hoc analysis, data were assessed for homogeneity of variance using Levene's test, as implemented in Origin 2023, the asterisks denote significant differences (^*^ *p* < 0.05,^***^ *p* < 0.001, n.s.—not significant).

The cytocompatibility of the developed hydrogels with MC3T3‐E1pre‐osteoblast cells was evaluated through an indirect assay after 48 h of incubation. Figure 9a shows that the presence of the phytotherapeutic hydrogels did not have an adverse effect on the cell viability when compared to the control group. Additionally, the incorporation of MBGNs+1.5CLV and MBGNs+3CLV in the hydrogels led to a notable increase in cell viability compared to the neat Alg‐Gel‐Xan hydrogel, thus suggesting the positive effect of both ion and CLV release on cell cytocompatibility. In all cases, the cell viability was above 80%, the value specified in the ISO‐10993‐5 standard as the non‐toxic threshold [78].

To gain insight into the cell morphology, fluorescence microscopy images were captured after 48 h of incubation (Figure 9 b1–b4). Fluorescence images of rhodamine‐phalloidin/DAPI staining demonstrated that the cell morphology was not affected by the presence of the hydrogels, with the cells maintaining their characteristic well‐spread spindle‐like shape. Furthermore, the cells exhibited well‐defined nuclei (blue) with the F‐actin filaments (red) being uniformly distributed within the cytoplasm, further confirming the biocompatibility of the hydrogels with MC3T3‐E1 pre‐osteoblast cells. These observations are in strong correlation with the cell viability results. In this approach, the cells were initially seeded in the well plate and allowed to reach confluency before exposing them to the products released from the hydrogel. Given that the cells were confluent before treatment and no significant differences in terms of cell morphology were observed after 48 h compared to the untreated group, other morphological changes at earlier time points are not expected.

Similar outcomes were reported by Ali et al. [79], who used lemongrass as a bioactive component in a chitosan/hydroxypropyl methyl cellulose/hydroxyapatite composite scaffold for BTE. The cytocompatibility tests conducted on the MC3T3‐E1 cell line revealed that increasing the concentration of lemongrass did not decrease cell viability. Even at higher concentrations, cell viability remained above 90% after 1 day, 3 days, and 7 days of testing. In contrast, when icariin was loaded into MBGNs and then incorporated into ADA‐Gel hydrogels, the cell viability decreased after 1 day in comparison with the control, as a result of the amount of active compound released [66]. Despite this decrease in viability, fluorescence microscopy images indicated that the materials still supported high‐density cell growth, with cells exhibiting good spread morphology on the surfaces. Surprisingly, for the icariin‐loaded samples, a change in the MC3T3‐E1 pre‐osteoblast cell morphology was observed. The cells had a cuboidal shape, which might suggest the differentiation of the cells without using specific differentiation factors.

## 3 Conclusion

The use of injectable hydrogels, known for their adaptability, biocompatibility, and capacity to fill irregular bone defects, represents a versatile and minimally invasive strategy with high potential in orthopaedics. In this study, we developed and characterized Alg‐Gel‐Xan hydrogels containing MBGNs loaded with two different concentrations of CLV. The investigations were focused on the morphological, mechanical, and chemical properties of these hydrogels, along with evaluating their antioxidant, antibacterial, and biocompatibility properties. The results revealed that all samples had a similar morphology, having a highly interconnected porous structure, needed for facilitating cell spreading and blood vessel formation. Moreover, ATR‐FTIR results confirmed the incorporation of MBGNs in the hydrogels, while the TPC further proved the presence of CLV in the hydrogels. The TPC analysis results were in line with the antioxidant activity measurements, demonstrating an enhanced antioxidant activity with increasing CLV concentration. Our findings also revealed that the addition of MBGNs+CLV did not compromise the injectability of the hydrogel formulations, as they could be easily and continuously extruded through a needle. In contrast, the addition of MBGNs+CLV enhanced the mechanical behaviour of the hydrogels. From a biological point of view, the MBGNs+CLV‐containing hydrogels had similar antibacterial effectiveness against *E. coli* and *S. aureus*, an effect that was enhanced by increasing the testing time. At the same time, all samples showed cytocompatibility with MT3C3‐E1 pre‐osteoblast cells, with a significant increase in cell proliferation for MBGNs+CLV samples. Fluorescence microscopy images showed well‐spread cells with a prominent nucleus, further confirming the material cytocompatibility. In summary, these findings underscore the potential of Alg‐Gel‐Xan‐MBGNs+CLV injectable hydrogels as an innovative and effective alternative for applications in bone regeneration strategies.

## 4 Experimental Section

### 4.1 Materials

The hydrogels were prepared using Alg (PH176) procured from JRS PHARMA GmbH& Co. KG (Rosenberg, Germany) and Xan obtained from Vitra2You (Fürth, Germany). Gel (type A, 300 g Bloom), sodium carbonate (Na_2_CO_3_, ≥99.5%, 223530), standard eugenol (≥99%, E51791), methanol (34860), and gallic acid (GAE, 27645) were acquired from Sigma‐Aldrich (Darmstadt, Germany). Microbial transglutaminase (mTG) was bought from Activa WM, Ajinomoto Foods Europe, Germany. 2,2‐diphenyl‐1‐picrylhydrazyl (DPPH, C_18_H_12_N_5_O_6_, D9132) was purchased from Biomol GmbH, Germany. Calcium chloride dihydrate (CaCl_2_•2H_2_O, 10035‐04‐8) and Folin–Ciocalteu's phenol reagent (FC, F9252) were obtained from Merck (Darmstadt, Germany). The antibacterial activity was performed on the *Escherichia coli* (ATCC25922) and *Staphylococcus aureus* (ATCC25923) microorganisms. Luria/Miller agar (LM, X969.1) and lysogeny broth medium (6673.1) were purchased from Carl Roth GmbH (Karlsruhe, Germany). The biological activity assay was carried out on the MC3T3‐E1 pre‐osteoblast cell line (99072810‐1VL) purchased from Sigma‐Aldrich (Darmstadt, Germany). Dulbecco's Phosphate Buffered Saline (DPBS, 10010023), Hank's Balanced Salt Solution (HBSS, no calcium, no magnesium, 88284), Dulbecco's modified Eagle's medium (DMEM, 31885‐023), penicillin/streptomycin (PS, 15140‐122), α‐minimum essential medium (α‐MEM, 22571038), 4′,6‐diamidino‐2‐phenylindole (DAPI, 62247), L‐glutamine (25030081), and rhodamine‐phalloidin (R415) were bought from Thermo Fischer Scientific (Schwerte, Germany). Additionally, WST‐8 assay (CCK‐8 Kit) and fetal bovine serum (FBS, F2442, Sigma‐Aldrich, Germany) from Sigma‐Aldrich (Darmstadt, Germany) were used.

### 4.2 Preparation of the Hydrogels

**FIGURE 10:**
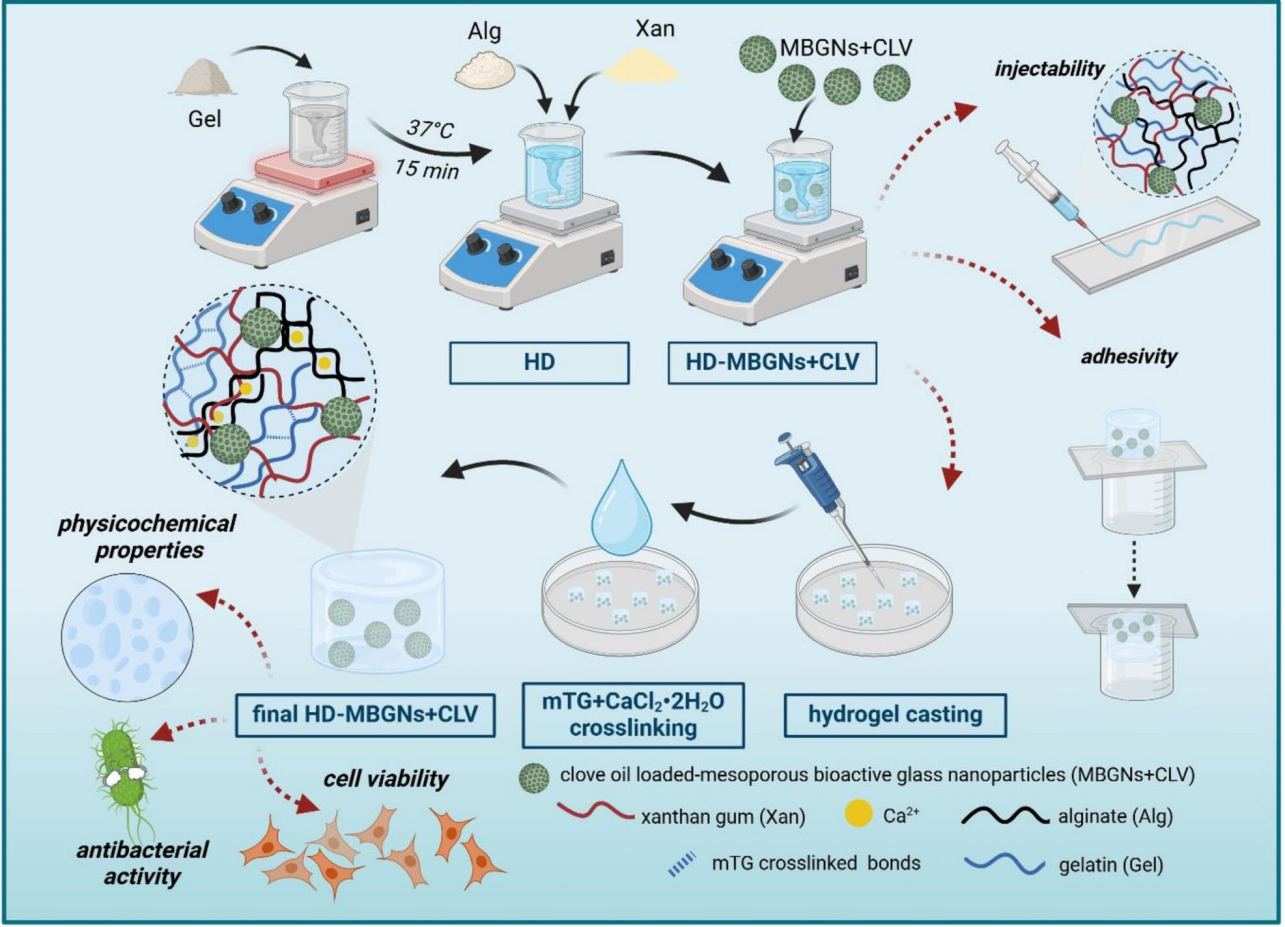
Schematic representation of the main preparation steps and characterization methods employed for the hydrogels. Created in BioRender. A., Damian (2025) https://BioRender.com/q7zsmsi.

Prior to hydrogel preparation, the CLV‐loaded MBGNs were obtained following a procedure reported by Damian‐Buda et al. [38]. The hydrogels were then produced by first dissolving Gel in DPBS at a content of 1% (w/v) for 30 min at 37°C. Subsequently, Alg and Xan were added to achieve final concentrations of 2.5% (w/v) and 1% (w/v), respectively. For the MBGNs+CLV hydrogels, the corresponding nanoparticles were also incorporated into the previously prepared solution at a concentration of 0.1% (w/v). After magnetic stirring the mixtures overnight, the hydrogels were cast into cylindrical molds (10 mm diameter, 4 mm height) and kept in the fridge for 5 min to induce physical gelation. Simultaneously, the crosslinking solution, containing 2.5% (w/v) mTG and 0.1 M CaCl_2_•2H_2_O, was prepared and filtered through 0.45 µm Millipore filters (Rotilab‐syringe filters, PVDF, Carl‐Roth). In the following step, the crosslinking solution was added on top of the hydrogel, left for 10 min, and subsequently washed with HBSS (Figure 10). In the end, three types of hydrogels were investigated: Alg‐Gel‐Xan, Alg‐Gel‐Xan containing MBGNs loaded with 1.5CLV (Alg‐Gel‐Xan‐MBGNs+1.5CLV), and Alg‐Gel‐Xan containing MBGNs loaded with 3CLV (Alg‐Gel‐Xan‐MBGNs+3CLV).

### 4.3 Morphological Characterization

The morphology of the hydrogels was characterized using a scanning electron microscope (SEM, AURIGA, Carl Zeiss). Before capturing the images, the cast and crosslinked hydrogels were frozen and lyophilized (Alpha 1–2 LD plus, Osterode am Harz, Germany). Subsequently, the dried samples were sputter‐coated with a thin layer of gold (Q150T Turbo‐Pumped Sputter Coater/Carbon Coater, Quorum Technologies). SEM images were analyzed with the ImageJ software (NIH, Bethesda, MD, USA) for measuring the diameter of 50 pores and calculating the average pore size for each sample.

### 4.4 Chemical Characterization

After casting and crosslinking the hydrogels, they were frozen and lyophilized. The dried hydrogels were then subjected to chemical analysis using attenuated total reflectance—Fourier‐transformed infrared spectroscopy (ATR‐FTIR) with an IRAffinity‐1S instrument from Shimadzu. Each spectrum was collected with a resolution of 4 cm^−1^, comprising 42 spectral scans, with the wavenumber being between 4000 and 400 cm^−1^, in absorbance mode.

### 4.5 Injectability and Adhesion Properties

The hydrogels' injectability, assessed prior to crosslinking, involved manually extruding the material through a 21 G needle using a 2‐mL syringe. Furthermore, the injection force required to extrude the hydrogels was quantified using a universal mechanical testing machine equipped with a 100 N load cell (Instron 5967, Instron GmbH, Germany) as proposed by Falcone et al. [80]. In this regard, the hydrogels were loaded into 3 mL syringes and fitted with 21 G needles. After fixing the syringe in the testing device, the injection force as a function of time was recorded at room temperature, with a speed of 5 mm/min for 120 s [50]. The average injection force was calculated from the plateau region of the resulting force‐time curves [51]. Each measurement was performed in five replicates, and the results were presented as the mean value ± standard deviation. At the same time, the adhesion properties of the uncrosslinked materials were examined by placing them on a glass plate, which was then inverted. The hydrogel behaviour was evaluated after 15 min.

### 4.6 Mechanical Properties

The mechanical properties of the Alg‐Gel‐Xan‐based hydrogels were assessed with a universal mechanical testing machine (Instron 5967, Instron GmbH, Germany). The hydrogels were prepared by casting them into moulds and crosslinking them according to Unalan et al. [16] and following the procedure described in detail in Section 2.2. Before conducting the measurements, the dimensions of the sample (diameter and height) were measured with a digital micrometer. Afterwards, a 100 N load cell at a strain rate of 1 mm/min was applied to each hydrogel sample at room temperature, with the resulting stress‐strain curves being further processed to find the modulus. Each type of hydrogel was tested in quadruplicate, with the results being presented in the form of the mean value ± standard deviation.

### 4.7 In Vitro Release and Degradation Studies

The in vitro CLV release study was performed by immersing five replicas for each hydrogel in 5 mL DPBS. At predefined testing intervals (1 day, 3 days, 7 days, 10 days, and 14 days), 1 mL supernatant was withdrawn and replaced with an equal volume of DPBS. To determine the cumulative release of CLV, the absorbance of the extract was measured at 281 nm with a UV–vis spectrophotometer (Specord 40, Analytik Jena GmbH, Jena, Germany). By employing a previously determined calibration curve of eugenol and the values of the absorbances, the concentration of CLV was further calculated.

The degradation study of the hydrogels was carried out by immersing five hydrogels of each type in 5 mL DMEM supplemented with 1% (v/v) PS. The weight evolution of the samples was monitored after 1 day, 3 days, 7 days, 10 days, and 14 days, and, based on the results, the swelling/degradation of the samples was quantified, as described by Equation (1):

(1) $$Swelling/degradation\;degree\;\left(\%\right)=\frac{w_{t}-w_{0}}{w_{0}}\;\times 100$$

where *w_t_* is the weight of the sample at the measuring time and *w_0_* is the initial weight of the sample.

### 4.8 Total Phenolic Content and Antioxidant Activity

TPC of Alg‐Gel‐Xan, Alg‐Gel‐Xan‐MBGNs+1.5CLV, and Alg‐Gel‐Xan‐MBGNs+3CLV was quantified through a slightly modified Folin–Ciocalteu (FC) assay [16]. In this regard, the FC solution was prepared by diluting the FC reagent nine times in deionised water (DW). Simultaneously, Na_2_CO_3_ was dissolved in deionized water at a concentration of 7.5% (w/v). After immersing the hydrogels in 2 mL of methanol for 24 h, 2 mL of FC solution and 4 mL of Na_2_CO_3_ were added to the methanol‐hydrogel solutions. The resulting mixtures were stored in the absence of light and at room temperature for 1.5 h before measuring the absorbance at 765 nm with a UV–Vis spectrophotometer (Specord 40, Analytik Jena GmbH, Jena, Germany). The TPC, expressed in mg of GAE/mg of sample, was determined using DW as a blank, according to Equation (2):

(2) $$TPC\;\left(mg\;of\;GAE\;per\;sample\right)=c\times V_{t}$$

where *c* is the concentration of GAE and *V_t_* is the total volume of the solution.

Besides TPC, the antioxidant activity of the samples was evaluated by carrying out a DPPH assay [40]. After immersing each sample in 2 mL of methanol for 3, 6, 24, 72, 120, and 144 h, 0.5 mL of the resulting extract was mixed with 2.5 mL DPPH radical solution, with the solution being further stored in the dark at room temperature for 1 h. The concentration of the DPPH solution was 0.04 mg/mL. To quantify the DPPH radical scavenging activity, the absorbance of the solutions was measured at 517 nm using a UV–Vis spectrophotometer (Specord 40, Analytik Jena GmbH, Jena, Germany). At the same time, the absorbances of the control (DPPH diluted with methanol) and blank (methanol) were recorded. To determine the DPPH radical scavenging activity, Equation (3) was employed:

(3) $$DPPH\;radical\;scavenging\;activity\left(\%\right)=\frac{A_{CNT}-A_{sample}}{A_{CNT}}\;\times 100$$

where *A_CNT_* and *A_sample_* are the absorbances of the control and sample, respectively.

### 4.9 Antibacterial Activity

To evaluate the antibacterial properties of the simple Alg‐Gel‐Xan hydrogels and the MBGNs‐containing samples, a turbidity assay was performed with *Escherichia coli* (Gram‐negative) and *Staphylococcus aureus* (Gram‐positive) bacterial strains. The experiments were carried out according to a previously reported work [16]. Prior to being in contact with the materials, the microorganisms were incubated in 10 mL LM at 37°C. After 24 h, the optical density (OD) of the suspension was measured at 600 nm and adjusted to 0.015 (Thermo Scientific GENESYS 30, Germany). Simultaneously, the samples were sterilized for 1 h on each side with UV irradiation, followed by immersion in 2 mL of LM for 24 h at 37°C. The following day, 20 µL of bacterial suspension was added to each sample. The resulting mixtures were further kept in the incubator at 37°C for 3, 6, and 24 h. At each pre‐determined timepoint, the OD value at 600 nm was measured (plate reader, PHOmo, Anthos Mikrosysteme GmbH, Germany). Additionally, bacterial suspension grown in the absence of the samples was used as a control, while LB medium was considered as blank. The relative bacterial viability was determined with Equation (4):

(4) $$Relative\;bacterial\;viability\;\left(\%\right)=\frac{OD_{sample}-OD_{blank}}{OD_{CNT}-OD_{blank}}\times 100$$

where *OD_sample_*, *OD_blank,_* and *OD_CNT_* are the optical densities of the sample, blank, and control, respectively.

In addition, the antibacterial efficacy of the hydrogels against *E. coli* and *S. aureus* was assessed using the colony counting assay following the procedure established previously [40]. After preparing the corresponding bacterial suspension by adjusting the OD to 0.015, the scaffolds were incubated in 2 mL of the suspension at 37°C for 24 h. Subsequently, serial dilutions (10⁴, 10⁵, and 10⁶) of the bacteria cultured in the presence of the material were prepared. Aliquots of 20 µL from each dilution were spread onto agar plates and incubated for another 24 h at 37°C. Afterwards, the number of colonies was quantified using the ImageJ software.

The morphology of *E. coli* and *S. aureus* bacterial strains was characterized using SEM [40]. Briefly, bacterial cells were fixed with 2.5% glutaraldehyde, followed by application of a series of ethanol/water solutions (from 30% to 99%) to dehydrate the samples. Subsequently, the samples were dried using a critical point dryer (Leica EM CPD300, Istanbul, Turkey), and SEM images of the dried samples were captured using an SEM microscope (AURIGA, Carl Zeiss).

### 4.10 In Vitro Cytocompatibility and Cell Morphology

For evaluating the in vitro MC3T3‐E1 pre‐osteoblast cell cytocompatibility of the hydrogel samples, an indirect contact method was employed (ISO‐10993‐5 standard). In this regard, the cells were exposed to the hydrogels’ dissolution products, followed by measuring the cell viability with a WST‐8 assay kit [78]. First, MC3T3‐E1 pre‐osteoblast cells were cultured in α‐MEM containing 10% FBS, 1% PS, and 1% L‐glutamine at 37°C in a humidified atmosphere (95% air and 5% CO_2_). When the cells reached 80% confluency, they were seeded in 24‐well plates at a seeding density of 1 × 10^5^ cells/well. Simultaneously, the hydrogels underwent sterilization for 2 h under UV irradiation. After UV treatment, the hydrogels were incubated in α‐MEM (10% FBS, 1% PS, and 1% L‐glutamine) at 37°C and 5% CO_2_ for 24 h to obtain the extract. The cell culture medium in which the cells were grown for 24 h was then replaced with the obtained extracts. The cells were further incubated in these conditions for 48 h, followed by quantifying the cell viability with the WST‐8 assay kit (1% (v/v)). More precisely, for each sample, the WST‐8‐cell culture medium solution was added and incubated in normal conditions for 3 h, when the absorbance of the WST‐8‐based solution was measured using a microplate reader. The control group consisted of the cells incubated in the absence of the hydrogel extract, and the WST‐8 solution served as a blank. Based on these measured values, the relative cell viability was calculated with Equation (5):

(5) $$Relative\;cell\;viability\;\left(\%\right)=\frac{A_{sample}-A_{blank}}{A_{CNT}-A_{blank}}\;\times 100$$

where*, A_sample_, A_blank,_ and A_CNT_* are the absorbances of the sample, blank, and control, respectively.

Following the 48‐h incubation period, rhodamine‐phalloidin and DAPI staining were used for visualizing actin filaments and nuclei, respectively. The staining procedure was done according to the manufacturer's specifications. In the first step, the cells were exposed to a 2.5% (v/v) paraformaldehyde solution for 45 min to ensure their fixation prior to the main staining procedure. Afterwards, the cells were incubated for 45 min at 37°C in an 8 µL/mL rhodamine‐phalloidin‐DPBS solution and finally for another 5 min at 37°C in the DAPI‐DPBS solution (1 µL/mL). After a final washing step with DPBS, the fluorescence images were captured with a DMI 6000B fluorescence microscope (Leica, Germany).

### 4.11 Statistical Analysis

The experiments were conducted in triplicate (if not otherwise stated), and the results were expressed as the mean value ± standard deviation (SD). Statistical analyses were performed using one‐way ANOVA, followed by post hoc Bonferroni tests, as implemented in Origin 2023 software (OriginLab, Northampton, MA, USA). Asterisk symbols (^*^ *p* < 0.05, ^**^ *p* < 0.01, and ^***^ *p* < 0.001) were employed for showing the levels of significant differences.

## Author Contributions

A.‐I.D.‐B. contributed to methodology, validation, formal analysis, investigation, data curation, writing—original draft, and writing—review & editing. M.L. contributed to methodology and formal analysis for mechanical testing, and reviewed and edited the original draft. A.R.B. was responsible for conceptualization, resources, writing—review, and editing, supervision, project administration, and funding acquisition. I.U. contributed to conceptualization, supervision, methodology, validation, investigation, formal analysis, data curation, writing—original draft, and review & editing.

## Conflicts of Interest

The authors declare no conflicts of interest.

## Data Availability

The data that support the findings of this study are available from the corresponding author upon reasonable request.
